# Evaluation of the clinical value of ELISA based on MPT64 antibody aptamer for serological diagnosis of pulmonary tuberculosis

**DOI:** 10.1186/1471-2334-12-96

**Published:** 2012-04-20

**Authors:** Changtai Zhu, Jinming Liu, Yang Ling, Hua Yang, Zhonghua Liu, Ruijuan Zheng, Lianhua Qin, Zhongyi Hu

**Affiliations:** 1 Department of Medical Laboratory, Changzhou Tumor Hospital Soochow University, Changzhou, 213001, China; 2 Shanghai Key Laboratory of Tuberculosis, Shanghai Pulmonary Hospital, Tongji University School of Medicine, Shanghai, 200433, China; 3 Department of Respiratory Medicine, Shanghai Pulmonary Hospital, Tongji University School of Medicine, Shanghai, 200433, China; 4 Shanghai Key Laboratory of Tuberculosis, Shanghai Pulmonary Hospital, Tongji University School of Medicine, No. 507 Zhengmin Rd, Shanghai, 200433, People's Republic of China

**Keywords:** MPT64 antibody, System evolution of ligands by exponential enrichment (SELEX), ssDNA, Aptamer, Tuberculosis

## Abstract

**Background:**

Presently, tuberculosis (TB) poses a global threat to human health. The development of reliable laboratory tools is vital to the diagnosis and treatment of TB. MPT64, a protein secreted by *Mycobacterium tuberculosis* complex, is highly specific for TB, making antibody to MPT64 a reagent specific for the diagnosis of TB.

**Method:**

Antibody to MPT64 was obtained by a combination of genetic engineering and immunization by the system evolution of ligands by exponential enrichment. A high-affinity aptamer of antibody to MPT64 was selected from a random single-stranded DNA library, and a sandwich ELISA method based on this aptamer was developed. This ELISA method was used to detect TB in 328 serum samples, 160 from patients with pulmonary TB (PTB) and 168 from non-tuberculous controls.

**Results:**

The minimum limit of detection of the ELISA method was 2.5 mg/L, and its linear range varied from 10 mg/L to 800 mg/L. Its sensitivity, specificity, positive likelihood ratio, negative likelihood ratio and area under the curve, with 95 % confidence intervals, were 64.4 % (56.7 %–71.4 %), 99.4 % (96.7 %–99.9 %), 108.2 (15.3–765.9), 0.350 (0.291–0.442) and 0.819 (0.770–0.868), respectively. No significant difference in sensitivity was observed between sputum smear positive (73/112, 65.2 %) and negative (30/48, 62.5 %) individuals.

**Conclusions:**

This sandwich ELISA based on an MPT64 antibody aptamer may be useful for the serological diagnosis of PTB, both in sputum smear positive and negative patients.

## Background

Being a contagious disease, tuberculosis (TB) poses a global threat to human health. The World Health Organization [1] reported that in 2010, there were 8.8 million (range, 8.5–9.2 million) incident cases of TB, 1.1 million (range, 0.9–1.2 million) deaths from TB among HIV-negative individuals and 0.35 million (range, 0.32–0.39 million) deaths from HIV-associated TB. In China, dramatic reductions in TB cases and deaths have been achieved. Although the Stop TB Partnership target of halving TB prevalence rates by 2015 compared with 1990 was unlikely to be achieved globally, this target had already been reached in the Americas and was close to being reached in the Western Pacific Region [1]. Two important factors have contributed to the resurgence and morbidity of TB: (i) the emergence of multi-drug-resistant and extensively drug-resistant strains of *Mycobacterium tuberculosis* and (ii) the increasing incidence of HIV globally [2-6]. Most patients with TB have pulmonary TB (PTB), and developing reliable laboratory tools is vital to the diagnosis and treatment of PTB [7].

Antibody to MPT64 has been shown to bind MPT64 protein [8], a highly specific protein secreted by *M. tuberculosis* (MTB) complex, which includes *M. tuberculosisM. bovis* and *M. africanum*[9-11]. Therefore, this antibody may be useful in the detection of TB. We describe here the use of the systematic evolution of ligands by exponential enrichment (SELEX) technique to develop a sandwich ELISA method based on an MPT64 antibody aptamer for the diagnosis of PTB.

## Materials and methods

### Ethics Statement

All patients were treated in accordance with the Helsinki Declaration on the participation of human subjects in medical research. The study protocol was approved by the Tongji University Ethics Committee (permit number: K08-018), and informed consent was obtained from each participant. Animal studies were approved by the Second Military Medical University Animal Care and Ethics Committee [approval ID: SYXK/2007-0003].

### Preparation of antibody to MPT64

Based on the sequence of the H37Rv TB reference strain encoding the MPT64 gene (accession number: NC_000962), we designed forward (5'-CCCATATGCGCATCAAGATCTTCAT-3') and reverse (5'-CCAAGCTTGGCCAGCATCGAGTCG-3') primers, containing Nde I and Hind III restriction sites, respectively. PCR amplification with these primers was performed using H37Rv nucleic acid as template. Subsequently, the purified PCR product was inserted into the T vector using ligase. After digestion with restriction enzyme, pMD18-MPT64 was to pET21a using T4 ligase and the pET21a-MPT64 expression plasmid was used to transform BL21 (DE3) competent cells. Positive recombinant clones were identified by DNA sequencing.

**Figure 1:**
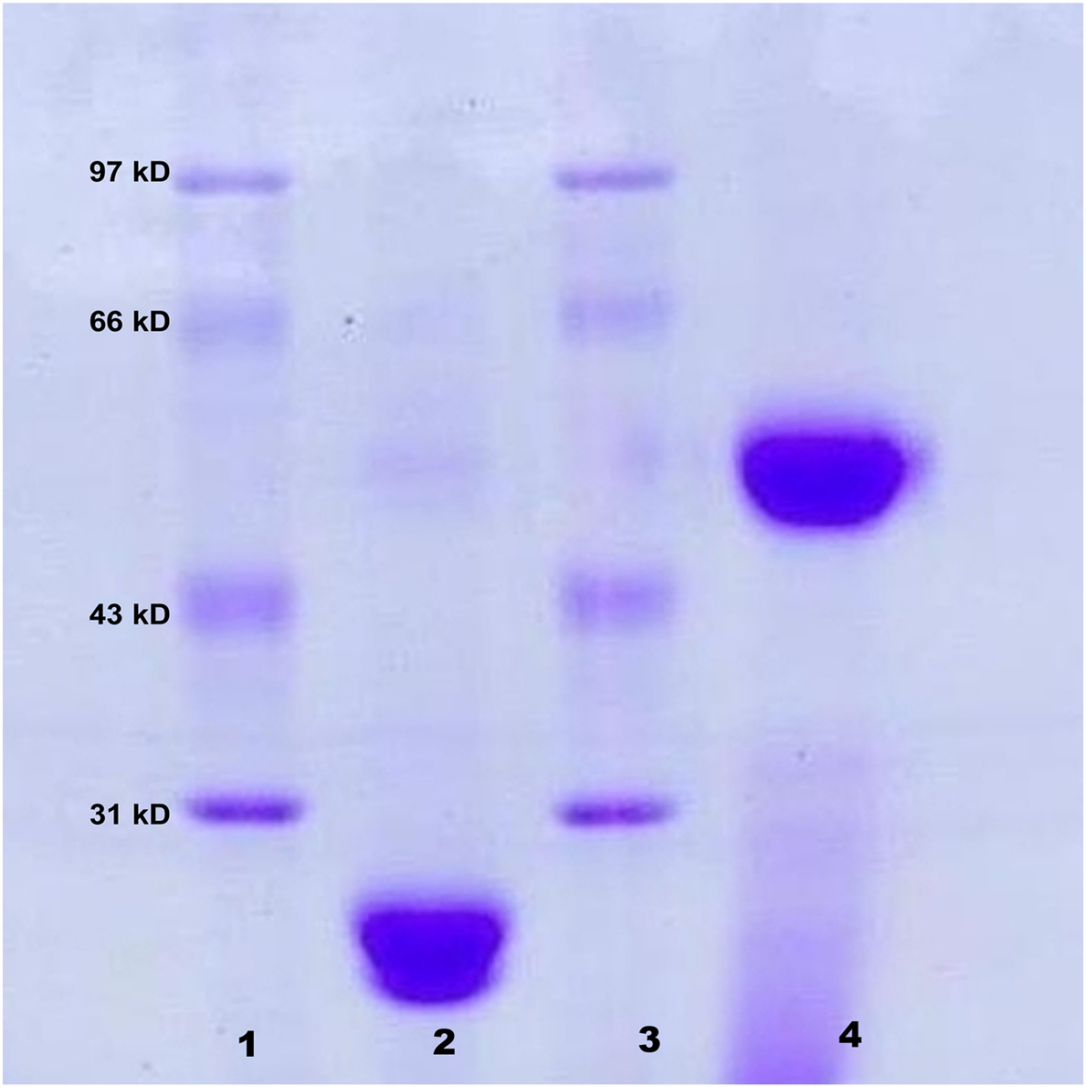
** SDS-PAGE analysis of MPT64 protein obtained by genetic recombination.** Lanes 1 and 3: markers; Lane 2: purified MPT64 protein (24 kD); Line 4: purified polyclonal antibody to MPT64 (50 kD).

A recombinant clone was inoculated overnight into the Luria Bertani medium at 37°C. The resultant bacteria were lysed with ultrasound and the medium was centrifuged. The sediment was resuspended in 10 % Triton-X 100 buffer, and MPT64 protein was purified on a nickel-agarose column. MPT64 protein was identified by sodium dodecyl sulfate polyacrylamide gel electrophoresis [12] [Figure 1.

**Figure 2:**
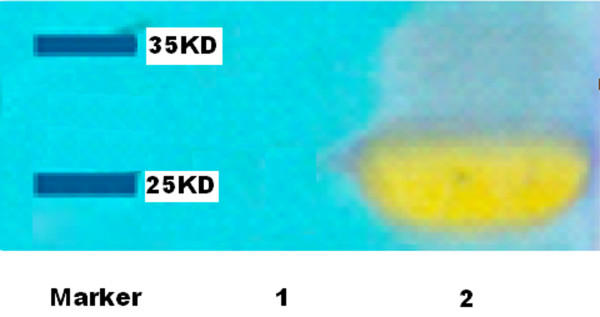
** Western blot for detecting MPT64 purified recombination protein ** Line 1: control (non-induced recombinant bacteria); Line 2: purified MPT64 recombination protein (24 kD).

Rabbits were immunized with MPT64 protein by subcutaneously injecting a suspension of 0.5 ml phosphate-buffered saline (PBS] containing 0.5 g/L MPT64 protein and 0.5 ml Freund's complete adjuvant into six sites on the back of each rabbit. On days 14, 24, 34, and 44, each rabbit was injected with the same dose of MPT64 protein and Freund's incomplete adjuvant. Rabbit serum was collected, and MPT64 antibody was purified on a CNBr-activated Sepharose 4B column, with the purified antibody identified by western blotting [Figure 2. The details of the method have been described previously [13]. Purified antibody was quantified by the Bradford method [14].

### Construction of a random single-stranded DNA (ssDNA) library

Using the primers 5’-GGGAGCTCAGAATAAACGCTCAA-3’ (forward) and 5’-Biotin-TTCGACATGAGGCCCGGATC-3’ (reverse), a 78-mer ssDNA library was constructed with the sequences, 5’ -GGGAGCTCAGAATAAACGCTCAA-AN35-CGACATGAGGCCCGGATC-3’, with the middle 35 sequences being random. The random ssDNA library and primers were synthesized commercially by Shanghai Sangon Company.

### Selection of MPT64 antibody aptamer

The ssDNA library was optimized by PCR amplification to obtain a double-stranded DNA (dsDNA) library. Furthermore, the ssDNA library was constructed by using asymmetric PCR. The amplified product was purified with a phenol-chloroform-isoamyl alcohol mixture (volume ratios, 25:24:1)]. ELISA plates coated with bovine serum were used to remove non-specific nucleotides from the ssDNA library and the purified ssDNA library added to microplates coated with antibody to MPT64. The plates were washed six times with SELEX buffer. SELEX eluate (20.0 mM Tris-HCl, 4.0 mM guanidinium isothiocyanate, 1.0 mM 1,4-dithiothreitol; pH 8.3) was added and allowed to incubate at 80°C for 10 min. The ssDNA was extracted with a phenol-chloroform-ethanol mixture. Using the extracted ssDNA as a template, asymmetric PCR was performed. Then, the next round of screening was performed. The parameters of the SELEX assay are shown in Table 1.

**Table 1:** Parameters during each round of the SELEX assay

**SELEX rounds**	**Antibody (μg/well)**	**ssDNA(μg/well)**	**Reaction time in BSA-blocked blank wells (min)**	**Reaction time in antibody-coated wells (min)**
1	1	0.5	15	60
2	0.5	0.5	20	60
3	0.25	0.5	20	60
4	0.25	0.1	30	45
5	0.1	0.1	30	45
6	0.25	0.1	30	45
7	0.1	0.1	45	30
8	0.1	0.05	45	30
9	0.05	0.05	45	30
10	0.05	0.025	60	20
11	0.025	0.025	60	20
12	0.025	0.01	60	15

After 12 rounds of aptamer selection, the absorbance of the ssDNA reached its maximum value and a single electrophoretic stripe was displayed. This indicated that the purified ssDNA library with high affinity to the anti-MPT64 antibody was saturated. The PCR products were purified using the TIANgen Midi Purification Kit (TIANgen Biotech [Beijing] Co., Ltd.) and subcloned into a pMD 18-T vector with a TA cloning kit (TaKaRa, Dalian, China). The resultant bacteria were used to transform *Escherichia coli* DH5α strains, and individual bacterial clones were selectively sequenced by a commercial company (Sangon, Shanghai, China).

The affinity of the selected ssDNA aptamers was measured by ELISA. Polystyrene microplates were coated overnight at 4°C with 10 μg/well of anti-MPT64 antibody in 100 μl 0.1 M NaHCO_3_ (pH 9.4), washed four times with washing buffer (PBS containing 0.05 % Tween 20, pH 7.4) and incubated for 1 hour at room temperature with 200 μl of blocking buffer. The microplates were washed once with washing buffer and 1.0 μg/well of biotin-labeled DNA aptamer in a binding buffer (SHCMK) containing 20.0 mM Hepes, pH 7.35, 1.0 mM CaCl_2_, 120 mM KCl and 1.0 mM MgCl_2_ was added. The aptamers and MPT64 antibody were allowed to react at 37°C for 40 min. The plates were washed six times with washing buffer (SHCMK + 0.05 % Tween 20) and incubated at 37°C for 30 min with 100 μl/well of streptavidin-horseradish peroxidase (Sigma, USA) diluted 1:1000 in PBS. Finally, the plate was washed six times with PBST and 100 μl of 1.0 mM tetramethylbenzidine in citrate buffer (0.1 M, pH 4.25), with 2.0 mM H_2_O_2_ added as substrate in a ratio of 1:20. The enzymatic reaction was stopped 5 min later by the addition of 50 μl 1.0 M H_2_SO_4_ and optical density (OD) of 450 nm was measured spectrophotometrically.

### Sandwich ELISA based on the anti-MPT64 antibody aptamer

We assessed the secondary structures of the aptamers using sequencing results and the DNAMAN version 4.0 (Lynnon Corporation, Quebec, Canada). The aptamer with highest affinity to the anti-MPT64 antibody was regarded as the capture and detection aptamer by ELISA. Each microplate well was coated with 0.1 μg of the capture aptamer, and the 5 ' end of the detection aptamer was labeled with biotin. Based on the detection of purified anti-MPT64 antibody at different dilution ratios, the minimum limit of detection and the linear range of this ELISA method were determined.

### Serum samples, participant characteristics and validation of the ELISA

We obtained 328 serum samples, 160 from patients with definite PTB and 168 from non-tuberculous controls including 78 healthy volunteers and 90 non-tuberculous patients. All participants were confirmed as being serologically HIV-negative. Of the 90 non-tuberculous patients, 35 had pneumonia, 40 had lung cancer, and 15 had lung abscesses and other conditions. Lowenstein-Jensen (L-J) culture was performed on all of these subjects to rule out TB.

The median ages of the PTB (31 years; range, 12–73 years; IQR [interquartile range], 17–45 years) and non-tuberculous controls (28 years; range, 14–69 years; IQR, 18–42 years) did not differ significantly *P* > 0.05). Patients were diagnosed with PTB by physicians based on the criteria of the Chinese Anti-tuberculosis Association, mainly including continuous fever and cough for more than 3 weeks, abnormal chest X-ray, weight loss and the demonstration of TB in sputum by L-J culture and standard biochemical identification tests. Sputum smear examinations for PTB and non-tuberculous patients were performed using the Ziehl-Neelsen method at the same time as L-J culture inoculation.

### Statistical analysis

The sensitivity, specificity, positive likelihood ratio (PLR), negative likelihood ratio (NLR) and area under the curve (AUC), each with 95 % confidence interval (CI), were calculated. All statistical analyses were performed using Stata version 9 (Statacorp, Texas, USA).

## Results

### Affinity, sequencing and secondary structure of ssDNA aptamers

**Figure 3:**
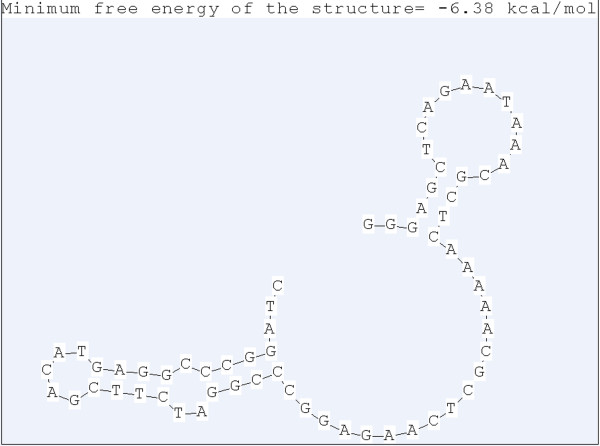
Secondary structure of the aptamer MPT64-A1 predicted by DNAMAN.

The OD values of ssDNA aptamers against MPT64 antibody (reflecting affinity) ranged from 0.55 to 1.62 [Table 2]. We elected MPT64-A1, which had a higher affinity (OD value: 1.62) than did the capture aptamer for ELISA. The sequences of aptamer included forward fixed sequences (5’- GGGAGCTCAGAATAAACGCTCAAA-3’), random sequences (5’- AACGCTCAAGAGGCCCGGATC-3’) and reverse fixed sequences (5’-TTCGACATGAGGCCCGGATC-3’). The DNAMAN package predicted that the secondary structure of MPT64-A1 is a stem-loop structure, similar to a large pocket [Figure 3].

**Table 2:** Affinities of ssDNA aptamers to anti-MPT64 antibody

**Name of aptamers**	**OD value**	**Name of aptamers**	**OD value**	**Name of aptamers**	**OD value**
MPT64-A1	1.62	MPT64-A5	0.93	MPT64-A9	1.24
MPT64-A2	0.82	MPT64-A6	0.55	MPT64-A10	1.12
MPT64-A3	0.78	MPT64-A7	0.90	MPT64-A11	1.10
MPT64-A4	0.96	MPT64-A8	1.33	MPT64-A12	1.06

### Minimum detection limit and linear range of this ELISA

**Figure 4:**
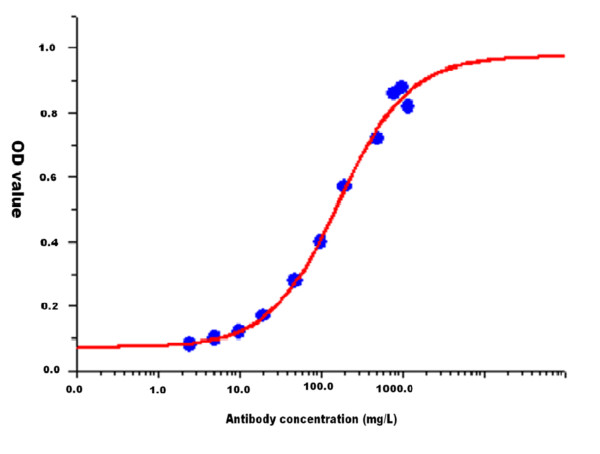
Linear range and minimum detection limit of the ELISA method.

Based on the detection of the purified MPT64 antibody at different dilution ratios, we found that the limit of detection of this method was 2.5 mg/L and the linear range varied from 10 mg/L to 800 mg/L [Figure 4]**.**

### Performance of the ELISA for serological diagnosis of PTB

In diagnosing PTB, this method had a sensitivity, specificity, PLR, NLR, and AUC, with 95 % CI, of 64.4 % (56.7 %–71.4 %), 99.4 % (96.7 %–99.9 %), 108.2 (15.3–765.9), 0.35 (0.291–0.442), and 0.819 (0.770–0.868), respectively [Table 3]. Its sensitivity was similar for sputum smear positive (73/112, 65.2 %) and negative (30/48, 62.5 %) individuals [Table 4].

**Table 3:** Performances of ELISA based on an anti-MPT64 antibody aptamer for serological diagnosis of pulmonary tuberculosis

		**PTB(n = 160)**	**Non-TB(n = 168)**	**Testing performances**				
				**Sensitivity(%, 95 % CI)**	**Specificity(%, 95 % CI)**	**PLR(95 % CI)**	**NLR(95 % CI)**	**AUC**
ELISA	Positive(n = 104)	103	1	64.4	99.4	108.2	0.358	0.819
	Negative(n = 224)	57	167	(56.7–71.4)	(96.7–99.9)	(15.3–765.9)	(0.291–0.442)	(0.770–0.868)

PLR: positive likelihood ratio. NLR: negative likelihood ratio. CI: confidence interval. PTB: pulmonary tuberculosis. AUC: area under the curve.

**Table 4:** ELISA based on an anti-MPT64 antibody aptamer for serological diagnosis of pulmonary tuberculosis with sputum smear positive and negative patients

		**Smear**		**χ**^**2**^	***p***** value**
		**Positive (n = 112)**	**Negative (n = 48)**		
ELISA	Positive (n = 103)	73	30	0.105	0.746
	Negative (n = 57)	39	18		

## Discussion

MPT64 protein is one of the main filtrate proteins secreted by MTB complex at an early stage. This protein is encoded by the Rv1980c gene, contains 228 amino acids and has a molecular mass of about 24,000 Da. It is a specific protein, being secreted only by the MTB complex [15-18]. In skin tests, this protein has a sensitivity of 87.8 % and a specificity of 100 % for diagnosing active TB [19,20]. Except for the MTB complex, bioinformatics showed no epitopes similar to MPT64 protein, indicating its uniqueness. Furthermore, few BCG vaccines express MPT64 protein [17], suggesting that anti-MPT64 antibody may be specific for the diagnosis of TB. Therefore, using a combination of immunization and genetic engineering with SELEX, we developed a sandwich ELISA method based on an anti-MPT64 antibody aptamer for the diagnosis of PTB.

SELEX [21,22] is a combinatorial chemistry technique used in molecular biology to produce single-stranded DNAs or RNAs that specifically bind ligands. SELEX can be used to select high affinity aptamers against target ligands and to obtain specific aptamers from an in vitro pool of random aptamers. SELEX methods have improved drastically in recent years, and several innovative SELEX approaches have been developed, including counter-SELEX, tissue-SELEX, cell SELEX (TECS-SELEX), fluorescent bead SELEX (FluMag-SELEX), capillary electrophoresis SELEX (CE-SELEX), neutral SELEX, and non-SELEX [23-31]. SELEX was recently used to develop a aptamers against counterpart target ligands, such as *Campylobacter jejuni*[32]; C-reactive protein [33]; heterogeneous nuclear ribonucleoproteins (hnRNPs) A1 [34]; *hepatitis C virus* envelope glycoprotein E, botulinum neurotoxin [35]; the carboxyl terminus of Kirsten rat sarcoma viral oncogene homolog (K-RAS) protein [36], adenosine [37] and others. Some aptamers against cancer-related proteins, such as platelet-derived growth factor, vascular endothelial growth factor (VEGF), human epidermal growth factor receptor 2, nuclear factor κB, tenascin-C and prostate-specific membrane antigen [24,38-40], have also been selected. Moreover, aptamers against whole cells, particularly cancer cells, have been selected [38,41-46]. The aptamers selected by SELEX were demonstrated to have potential clinical value [47-50]. For example, pegaptanib (Macugen), used to treat neovascular age-related macular degeneration, and the anti-VEGF aptamer pegaptanib used to treat human ocular vascular disease, have received approval by the US Food and Drug Administration [51,52]. In summary, SELEX has attracted widespread attention and the selected aptamers have been used in clinical diagnosis, treatment and drug development.

Functionally, an aptamer is similar to a protein antibody complex obtained following immunization. Among the advantages of aptamers over classical protein antibody complexes are (1) higher specificity and affinity; (2) its binding to a greater variety of target ligands, including biological macromolecules, small molecule organic compounds, inorganic ions, pathogenic microorganisms, and cells; (3) increased convenience and lower cost; and (4) greater stability, including storage at room temperature.

We have utilized the SELEX method to obtain aptamers against anti-MPT64 antibody in order to develop an ELISA method for the diagnosis of TB. Following 12 rounds of screening, we observed a single electrophoretic strip, indicating that the purified ssDNA library with a high affinity for anti-MPT64 antibody was saturated.

However, we observed differences in the affinities of the selected aptamers. Its OD showed that MPT64-A1 had the highest affinity of all aptamers, whereas DNA sequencing showed that absorbances for detecting affinity were relatively constant. We found only small differences among the absorbance values for aptamers with single base differences at the 5', whereas differences in nucleotides at the stem-loop structure at the 3' end had significant effects on affinity to anti-MPT64 antibody. This suggested that the stem-loop structure may be the site at which aptamers bind to the MPT64 antibody. The secondary structure predicted by DNAMAN indicated that MPT64-A1 could form a stem-loop structure like a large pocket.

We found that the limit of detection of our ELISA method was 2.5 mg/L, with a linear range varying from 10 mg/L to 800 mg/L. This suggested that this method was sensitive and could have potential clinical value. ELISA had a high specificity (99.4 %, 95 % CI: 96.7 %–99.9 %) and PLR 108.2 (15.3–765.9) and was appropriate for diagnostic purposes. However, this method had a sensitivity of 64.4 % (95 % CI: 56.7 %–71.4 %) and an NLR of 0.350 (95 % CI: 0.291–0.442), suggesting that this method was not suitable for screening purpose. The AUC (0.819, 95 % CI: 0.770–0.868) showed the reliability of this ELISA method. In addition, the similar sensitivities observed in sputum smear positive and negative patients suggested that this ELISA method may be suitable for the serological diagnosis of sputum smear negative individuals. Since sandwich ELISA is a rapid, simple and very common test used by many laboratories and laboratory staff [53], it should prove feasible and useful in resource-limited settings.

However, we did not include subsets of patients infected with HIV/TB [54], indicating that the clinical value of this ELISA method for a HIV-endemic population remains unknown. Moreover, this study involved only a few specific populations from hospital settings. Therefore, further studies in different and more extensive populations are necessary to evaluate the power of this test for the diagnosis of TB.

## Conclusions

Using a combination of genetic engineering with SELEX and rabbit immunization, we developed a novel sandwich ELISA based on an anti-MPT64 antibody aptamer. Clinical validation showed that this ELISA method was a reliable test for the serological diagnosis of PTB and could be used especially for diagnostic purposes. However, this ELISA method could also be suitable for serological diagnosis of sputum smear negative populations.

## Competing interests

The authors declare that they have no competing interests.

## Authors’ contributions

CTZ, ZYH, LHQ and JML designed the study. CTZ, HY, ZHL, RJZ and LHQ performed the experiments. CTZ, JML and YL performed the statistical analysis. CTZ, LHQ and YL wrote the manuscript. JML, HY and ZYH provided valuable insight for revising the manuscript. All authors contributed to the study and have read and approved the final manuscript. ZYH is the guarantor.

## Pre-publication history

The pre-publication history for this paper can be accessed here:

http://www.biomedcentral.com/1471-2334/12/96/prepub
