# Correction: Evaluation of the clinical value of ELISA based on MPT64 antibody aptamer for serological diagnosis of pulmonary tuberculosis

**DOI:** 10.1186/1471-2334-13-410

**Published:** 2013-09-03

**Authors:** Changtai Zhu, Jinming Liu, Yang Ling, Hua Yang, Zhonghua Liu, Ruijuan Zheng, Lianhua Qin, Zhongyi Hu

**Affiliations:** 1 Department of Medical Laboratory, Changzhou Tumor Hospital Soochow University, Changzhou 213001, China; 2 Shanghai Key Laboratory of Tuberculosis, Shanghai Pulmonary Hospital, Tongji University School of Medicine, Shanghai 200433, China; 3 Department of Respiratory Medicine, Shanghai Pulmonary Hospital, Tongji University School of Medicine, Shanghai 200433, China; 4 Shanghai Key Laboratory of Tuberculosis, Shanghai Pulmonary Hospital, Tongji University School of Medicine, No. 507 Zhengmin Rd, Shanghai 200433, People’s Republic of China

## 

ssDNA pools 5′-GGGAGCTCAGAATAAACGCTCAA-AN35- CGACATGAGGCCCGGATC-3′ and reverse primer 5′-Biotin-TTCGACATGAGGCCCGGATC-3′ were reported incorrectly in the original version [1].

These have been corrected to ssDNA pools 5′-GGGAGCTCAGAATAAACGCTCAA-N35-TTCGACATGAGGCCCGGATC-3′ and reverse primer, which was complementary to the 3′ flanking sequence of the library template, 5′-Biotin-GATCCGGGCCTCATGTCGAA-3′.

This revision only corrects the description of ssDNA pools and reverse primer, which were incorrectly transcribed in the original manuscript, and has no effect on the results and conclusions of the original article [1].

## Pre-publication history

The pre-publication history for this paper can be accessed here:

http://www.biomedcentral.com/1471-2334/13/410/prepub
