# Correction: Evaluation of the Clinical Value of ELISA based on MPT64 Antibody Aptamer for Serological Diagnosis of Pulmonary Tuberculosis

**DOI:** 10.1186/1471-2334-13-430

**Published:** 2013-09-12

**Authors:** Changtai Zhu, Jinming Liu, Yang Ling, Hua Yang, Zhonghua Liu, Ruijuan Zheng, Lianhua Qin, Zhongyi Hu

**Affiliations:** 1 Department of Medical Laboratory, Changzhou Tumor Hospital Soochow University, Changzhou 213001, China; 2 Shanghai Key Laboratory of Tuberculosis, Shanghai Pulmonary Hospital, Tongji University School of Medicine, Shanghai 200433, China; 3 Department of Respiratory Medicine, Shanghai Pulmonary Hospital, Tongji University School of Medicine, Shanghai 200433, China; 4 Shanghai Key Laboratory of Tuberculosis, Shanghai Pulmonary Hospital, Tongji University School of Medicine, No. 507 Zhengmin Rd, Shanghai 200433, People's Republic of China

## 

ssDNA pools 5′-GGGAGCTCAGAATAAACGCTCAA-AN35- CGACATGAGGCCCGGATC-3′ and reverse primer 5′-Biotin-TTCGACATGAGGCCCGGATC-3′ were reported incorrectly in the original version [1].

These were corrected as ssDNA pools 5′-GGGAGCTCAGAATAAACGCTCAA-N35-TTCGACATGAGGCCCGGATC-3′ and reverse primer, which was complementary to the 3′ flanking sequence of the library template, 5′-Biotin-GATCCGGGCCTCATGTCGAA-3′.

These revises only corrected the error description of ssDNA pools and reverse primer, and have no effect on the results and conclusions of the original version [1].

## Pre-publication history

The pre-publication history for this paper can be accessed here:

http://www.biomedcentral.com/1471-2334/13/430/prepub
